# Multiple dimensions of uncertainty in fertility goals: recent trends and patterns in the United States

**DOI:** 10.1186/s41118-025-00251-6

**Published:** 2025-06-02

**Authors:** Luca Badolato, Sarah R. Hayford, Karen Benjamin Guzzo

**Affiliations:** 1 Ohio State University, 238 Townshend Hall, 1885 Neil Ave, Columbus, OH 43210 USA; 2 University of North Carolina at Chapel Hill, 155 Hamilton Hall CB3210, Chapel Hill, NC 27599 USA

**Keywords:** Fertility goals, Fertility intentions, Uncertainty, U.S. fertility, Fertility trends

## Abstract

A growing body of fertility research focuses on uncertainty as a key contributor to fertility decision making and behaviors. In this paper, we identify and describe multiple components of uncertainty in fertility goals that, when analyzed together and in relation to macro-level trends, provide critical insight into fertility dynamics. Drawing from multiple streams of research on fertility goals and behaviors, we focus on (i) *goal uncertainty*, (ii) *realization uncertainty*, and (iii) *intensity of goals*. We use data from the National Survey of Family Growth (NSFG) 2002–2019 to estimate trends and patterns of these three dimensions of uncertainty in fertility goals, with a focus on variation across the life course and inequality by education and income. We link uncertainty in fertility goals with the quantum and timing of fertility intentions among U.S. women. The results show that realization uncertainty is pervasive, with up to 50% of women who intend children being uncertain whether they will actually follow through with those intentions, and intensity of intentions is low, with up to 25% of childless women who intend children saying that they would not be bothered if they did not have a child. Although goal uncertainty is overall stable across the study period, young and childless women show increasing realization uncertainty over time and hold their positive intentions less intensely. More socioeconomically advantaged women exhibit higher realization certainty and greater intensity of their goals. Women who are more certain of realizing their positive intentions and those who hold them more intensely report a higher number of additional intended children and a shorter time frame for future childbearing. We conclude by situating these findings in a broader climate of increasing uncertainty.

After a period of stability around 2.0 children per woman in the 1990s and early 2000s, the U.S. Total Fertility Rate (TFR) reached a peak of 2.12 in 2007 (Human Fertility Database 2024) (Fig. 1). The TFR then steadily declined in the aftermath of the Great Recession, falling to 1.62 in 2023 (Martin, Hamilton, and Osterman 2024; Osterman et al. 2022). Cumulating evidence indicates that the decline in observed fertility cannot be entirely explained by a decline in overall fertility goals (an umbrella term encompassing ideals, desires, intentions, and the like), but fertility postponement and a decreased likelihood of achieving early-life fertility goals may play a large role (Guzzo & Hayford, 2023; Hartnett & Gemmill, 2020).

**Fig. 1:**
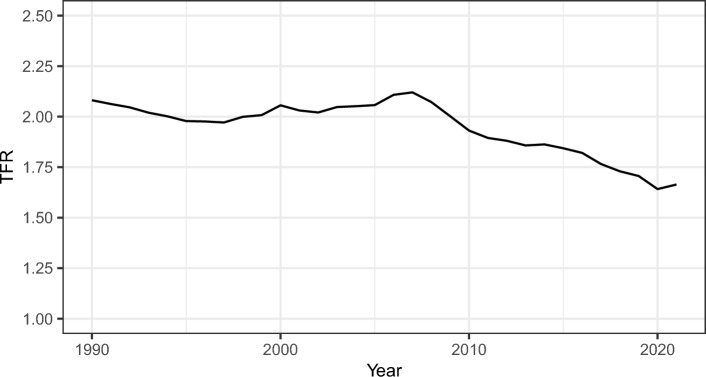
U.S. Total Fertility Rate 1990–2021 (Source: Human Fertility Database 2023)

Increased uncertainty around fertility goals may also contribute to declining birth rates. A growing body of research recognizes that individuals might have uncertain, mixed, or ambivalent feelings about their future childbearing (Bernardi, Mynarska, and Rossier 2015; Morgan, 1981, 1982; Ní Bhrolcháin and Beaujouan 2012; Schaeffer & Thomson, 1992). Those with uncertain goals may be less likely to carry them out, or might desire or intend fewer children, than those whose goals are more clear. However, the nature of uncertainty in fertility goals, the degree to which uncertainty has changed over time, and variation in uncertainty are not well understood. In addition, the relationship between the uncertainty in overall fertility goals and the specifics of fertility goals—how many children and when—is unclear. Less certainty about overall goals, especially when linked with behavioral aspects such as lower quantum goals and longer potential timeframes to achieve those goals—could be a crucial part of the story of declining contemporary fertility.

In this paper, we use data from the National Survey of Family Growth (NSFG), the most comprehensive nationally representative repeated cross-sectional survey on fertility goals, attitudes, and behaviors in the United States, to provide a broad overview of trends and patterns of uncertainty in fertility intentions before and after the Great Recession among U.S. women (from 2002 to 2018). Our contributions are threefold. First, we estimate overall levels and trends in three dimensions of uncertainty related to fertility goals: (i) uncertainty in goals; (ii) uncertainty about the realizability of goals; and (iii) intensity of goals. Second, we examine variation in levels and trends across life course factors (parental status, age, partnership status) and socioeconomic resources (education and household income). Third, we assess whether and how realization uncertainty and intensity are associated with the intended quantum (how many children) and timing (when to have children) of fertility, net of several sociodemographic characteristics. These descriptive analyses address the question of whether macro-trends in fertility levels correspond with macro-trends in uncertainty; at what stages and for whom uncertainty is most prevalent; and how overall uncertainty begins to be translated to behavior. Our analyses serve as a foundation for future analyses of uncertainty in fertility goals that more directly address the relationship between macro-level social and economic conditions and individual fertility decisions.

## Theoretical and empirical perspectives on uncertainty and fertility

In the last few decades, a growing body of fertility research across high-income countries has considered uncertainty following the Great Recession as a driver of declining fertility (Comolli, 2017; Sobotka et al., 2011). This literature examines the relationship between macro-level uncertainty, uncertainty in individuals’ lives, and fertility behaviors (Comolli & Vignoli, 2021; Currie & Schwandt, 2014; Manning et al., 2022; Min & Taylor, 2018; Schneider, 2015). The core argument of this body of research is a macro–micro framework according to which the instability and unpredictability of economic conditions, both at the macro-level and as experienced by individuals, make it more difficult to plan for children, thus reducing goals for future fertility and birth rates.

Macro-level uncertainty influences uncertainty in individuals’ lives across financial (i.e., unemployment, economic hardship) and nonfinancial domains (i.e., relationship instability, health concerns, fear of the future). At the individual level, this type of uncertainty has been linked to family dynamics, including union formation (Bolano & Vignoli, 2021), union dissolution (Di Nallo et al., 2022; Solaz et al., 2020), and childbearing (Currie & Schwandt, 2014; Ivanova & Balbo, 2024; Manning et al., 2022; Min & Taylor, 2018). Macro-level uncertainty has also played an important part in recent theorizing about fertility and analyses of individual-level fertility. For example, in the “Narratives of the Future” framework, rising economic uncertainty is described as a “game-changer for European fertility dynamics” (Vignoli et al., 2020, p. 4). In this framework, both objective economic uncertainty grounded in precarious employment and perceived economic uncertainty grounded in the perception of unstable futures negatively affect fertility intentions and behaviors.

A relatively smaller body of research focuses on uncertainty in fertility goals themselves. There is a growing understanding that prospective fertility goals are more complex than the binary yes/no constructs usually collected in large-scale fertility surveys and that individuals might be uncertain about their fertility goals in the first place. Morgan (1981, 1982), analyzing fertility intentions of U.S. women in the late 1970s, showed that “don’t know” answers to fertility intentions questions are meaningful responses and systematically associated with individuals’ characteristics. Building on this work more than three decades later, Bernardi et al. (2015), in their qualitative research conducted across European countries, identified four categories of uncertain fertility intentions: (i) *contingent* intentions (a child as a project, but prevented by various external obstacles such as lack of a partner or unemployment), (ii) *indifferent* intentions (childbearing never considered explicitly), (iii) *ambivalent* intentions (both positive and negative intentions), and (iv) *far* intentions (a child as a project, but in a later future).

There is no consensus on how to measure the uncertainty of fertility goals, in part because fertility questions are not harmonized across fertility surveys, and scholars often have to adapt their analyses to the measures available in secondary data. Some surveys include pre-coded questions that contain uncertain categories (such as “probably yes” and “probably no” intentions), while others only contain a “don’t know” option. Ní Bhrolcháin and Beaujouan (2012) show that levels of uncertainty vary substantially depending on which categories of uncertainty are included as possible answers and how uncertainty is defined. The use of different constructs across fertility surveys, including fertility desires, intentions, expectations, and ideals, is a further source of confusion (Badolato, 2025; Kost & Zolna, 2019; Miller, 2011).

Although interconnected in principle, these two streams of research about uncertainty at the macro and individual levels and about uncertainty as a measurement issue are rarely put in conversation. It would be logical to expect that uncertain conditions could produce less certain intentions as well as lower intentions, or that multiple dimensions of uncertainty could be connected. A few notable studies examine these issues. Research on perceptions of the HIV epidemic and fertility intentions and behaviors across sub-Saharan Africa has been central to theorizing how individuals organize their childbearing plans in uncertain contexts (Hayford et al., 2012; Trinitapoli, 2023; Trinitapoli & Yeatman, 2011). In these contexts, fear of the health implications of childbearing in combination with persistent pronatalist norms creates uncertainty in fertility plans, with some women wanting to stop while others want to accelerate childbearing. Similarly, Frye and Bachan (2017) show that non-numeric responses to fertility goals questions, such as “don’t know” or “up to God,” are not strict measurement issues but are influenced by several social factors. More broadly, Timaeus and Moultrie (2008) argue that uncertainty about the future is a pervasive force driving fertility in sub-Saharan Africa and call for a new approach to conceptualizing and operationalizing fertility desires that reflects this uncertainty. Across high-income countries, Comolli (2023) shows that changes in broader social climate and social uncertainty are associated with “don’t know” responses about fertility intentions.

## Multiple dimensions of uncertainty

In this paper, we describe levels, trends, and variation in multiple dimensions of uncertainty in fertility goals and analyze the relationship between these dimensions of uncertainty and the timing and quantum of prospective fertility goals.

First, we consider uncertainty about goals themselves, *goal uncertainty—*that is, do individuals find it difficult to form certain fertility goals? Second, we consider uncertainty about realizing those goals, *realization uncertainty*—that is, do people report plans to have (or not have) children but feel like they may not achieve their plans? Finally, we consider a concept related to, but distinct from, uncertainty: the *intensity* of intentions. Here, we suggest that while individuals may provide an answer about fertility goals when asked, their responses may not reflect firmly held plans. Put differently, even though individuals may report that they intend to have a child, their reported answers may not reflect deeply held convictions to do so or strong feelings about achieving these goals.

The first type of uncertainty, *goal uncertainty*, has received most attention in previous research, largely through analyses of “don’t know” answers (e.g., Comolli, 2023; Kuhnt et al., 2021) or “probably yes” and “probably no” answers (e.g., Barker & Buber-Ennser, 2024) to questions about fertility intentions.

The second type, *realization uncertainty*, has received less attention. Risk assessment is a central component of life planning and a poignant feature of modern societies (Beck, 1992; Giddens, 1991) and explicitly embedded in leading theories of fertility goals. In the narrative framework, *imagined futures*, including expectations and risk assessment of future life trajectories based on current and past experiences, are central to forming fertility intentions (Vignoli et al., 2020). Similarly, *perceived behavioral control*, including the perceived barriers affecting the likelihood of achieving fertility intentions, is central to the Theory of Planned Behavior (Ajzen, 1991; Ajzen & Klobas, 2013). Empirical research on fertility intentions uncertainty tends to categorize individuals into *certain* and *uncertain* categories of fertility intentions (focusing on goal uncertainty only) without recognizing that individuals who report *certain* positive or negative fertility intentions might be *uncertain* about their realizability. This distinction is relevant. Using cross-sectional data across European countries, Testa and Gietel-Basten (2014) show that the perceived economic downturn following the Great Recession was not associated with individuals’ fertility intentions but was associated with their certainty of meeting positive intentions. This dimension is also important in contexts of rising unrealized fertility and mismatches between fertility goals and behaviors (Casterline & Han, 2017; Guzzo & Hayford, 2023; Harknett & Hartnett, 2014; Morgan & Rackin, 2010).

The third type, *intensity* of goals, is related to, but slightly distinct from, uncertainty. This dimension relates fertility goals with other childbearing attitudinal measures, which are important to understanding fertility goals and behaviors (Aiken et al., 2016; Guzzo et al., 2019; Shepherd & Marshall, 2019). As Hin et al. (2011) note, fertility surveys rarely ask about the strength of people’s stated fertility goals. Although surveys may ask people to report their fertility intentions, it should not be assumed that people would be equally unwilling to deviate from their stated goals; it is possible that people give an answer because they are expected to (or, in the case of many surveys, forced to), but have actually mixed feelings and attitudes about their goals. The few instances in which respondents’ fertility goals can be compared to how strongly they feel about their goals bear out the idea that these mixed feelings are common (van Tintelen and Stulp 2024).

Different forms of uncertainty and intensity, in turn, are likely linked to the timing and quantum of fertility intentions. Bernardi et al. (2015) identified *far* intentions—a child as a project but far in the future—as uncertain intentions. Ní Bhrolcháin and Beaujouan (2012) included individuals who intended more children but not for at least three more years in one of their operationalizations of fertility intentions uncertainty. These studies suggest that those who are more uncertain and attached less intensely to their fertility goals tend to consider a longer time frame for childbearing and, possibly, a lower number of children. (The reverse process is also possible: those who intend children farther in the future may be less certain about their goals, if they anticipate changing life circumstances, and those who intend children in the near future may be more certain about their goals, if they already positively evaluated future circumstances.) This is a key mechanism linking macro-level uncertainty and fertility decline through the uncertainty of fertility goals: in contexts of macro-level uncertainty, individuals might find it more difficult to form certain fertility intentions or follow through with their intentions, postponing childbearing and considering fewer additional children. Still, no systematic study assesses whether uncertainty and the intensity of intentions are associated with the reported quantum and timing of fertility intentions, yet more specific fertility intentions indicators provide important information about likely future trends. Short-term fertility intentions are more predictive of actual fertility (Dommermuth et al., 2015), and quantum intentions are, in the U.S., the basis for fertility rate projections (Social Security Administration, 2024). Thus, understanding how overall goals, and different types of goal uncertainty, are related to quantum and timing intentions could shed light on actual fertility levels in the short term.

Any conceptual framework on fertility uncertainty would be incomplete without considering that uncertainty may not be static across the life course or uniform across subgroups (Bachrach and Morgan 2013; Guzzo & Hayford, 2020). As Rackin and Bachrach (2016) argue, fertility intentions become more crystalized (and thus more predictive of subsequent fertility behavior) when individuals occupy key statuses related to fertility, such as being partnered or when individuals are already parents. Similarly, fertility goals measured at younger ages may reflect internalization of abstract ideals, whereas those at older ages may capture more reasoned decision-making processes (Bhrolcháin and Beaujouan 2019). In addition, given the centrality of economic uncertainty in theories and uncertainty and fertility and our focus on trends before and after the Great Recession, it is important to analyze the role of socioeconomic status characteristics. The availability of resources, or anticipated future availability of resources, is a key predictor of desires and intentions to have children as intensive parenting models have become pervasive and the income demands of parenthood have grown (Ishizuka, 2019; van Wijk & Billari, 2024). Accordingly, we would expect people with more education and income to be more certain about their fertility goals because they are more likely to be confident in their assessment of their imagined futures (Suckert, 2022). In addition to its association with income and future income, education is also independently associated with a stronger sense of agency and control (Mirowsky & Ross, 2007).

## The context: socioeconomic uncertainties in the U.S.

Our analyses are situated in a complex context of socioeconomic uncertainty that evolved throughout and after the Great Recession. Although we do not directly analyze the relationship between economic conditions and fertility goals, it is important to contextualize our analyses in terms of broader economic conditions.

In the United States and elsewhere, the Great Recession had longstanding consequences and is recognized as a driver of fertility decline (Comolli, 2017; Goldstein et al., 2013; Matysiak et al., 2021). As highlighted by Schneider (2015, p. 1148), “the Great Recession served not only to increase realized hardship but also to generate feelings of economic uncertainty, insecurity, and fear.” As previous research in the U.S. showed in detail, objective and subjective economic indicators and structural economic changes, as well as generalized uncertainty about the future, have significantly affected fertility rates and achieved fertility (Comolli, 2017; Guzzo & Hayford, 2023; Schneider, 2015; Seltzer, 2019). Importantly, the impact of the Great Recession on fertility is not homogeneous across population subgroups: it is particularly strong among younger (Cherlin et al., 2013; Comolli, 2017; Schneider, 2015), childless (Comolli & Bernardi, 2015), and Hispanic individuals (Seltzer, 2019). Variation by socioeconomic status is less well-described, but some evidence suggests that declines in fertility in the post-Great Recession years were larger for less educated women than for other groups (Kearney et al., 2022). However, it is still unclear whether multiple dimensions of uncertainty in fertility goals changed throughout the Great Recession, and whether these changes occurred differently across population subgroups.

## Data and methods

This study is based on the National Survey of Family Growth (NSFG), which has collected nationally representative repeated cross-sectional data in the United States since 1973 to produce national estimates of a broad range of fertility-related indicators. Starting in 2006, the NSFG implemented a continuous interviewing design with data releases every two or four years. There are some important limitations in the male questionnaire. In particular, unpartnered men (not cohabiting and not married according to the NSFG definition) are not asked about the certainty of their fertility intentions. We therefore restrict the analyses to women only.^1^ The NSFG provides sample weights to adjust for survey design, nonresponse bias, and declining response rates across years, which can be used to compute aggregate representative statistics. Given our interest in computing recent trends and patterns of fertility intentions and childbearing attitudes in the aftermath of the Great Recession, we use the data from 2002 to 2019, which, when weighted, allows computing nationally representative statistics for 2002, 2008, 2012, 2014, 2016, and 2018 for an analytical sample of 41,492 women aged 15 to 44.

### Goal uncertainty, realization uncertainty, and intensity of goals

In the NSFG, questions about fertility goals follow a stepwise design. First, respondents are asked whether they *desire* more children: “If it were possible, would you, yourself, want to have (a/nother) baby at some time in the future?” Women without a coresidential different-sex partner who respond affirmatively and women in a cohabiting or marital different-sex partnership, regardless of their desires, are then asked if they *intend* a(nother) child; this is only asked if the respondent (and their partner) is physically able to have children.^2^ Specifically, they are asked “Do you (and [name of current married or cohabiting partner] if partnered) intend to have (a/nother) baby at some time (in the future/after this pregnancy is over)?” Importantly, women without a cohabiting or marital partner are asked about their individual fertility intentions, while partnered women are asked about their and their partners’ joint fertility intentions. Possible answers are “yes” or “no.” It is important to note that the NSFG does not directly offer to respondents the possibility of reporting “don’t know” about fertility intentions as a provided option. Instead, as a prompt available in the NSFG questionnaire and likely read to respondents who hesitate to answer, surveyors are instructed “If necessary, say: Intend refers to what you [and your husband/partner] are actually going to try to do.” In contrast to other questions in the same section, including the intended number of additional children where interviewers are instructed not to probe “don’t know” answers because follow-up questions are available in those cases, no direct instructions are included in fertility intentions, and the NSFG records responses of “don’t know” as well as refusals. (For brevity, we refer to these combined groups as “don’t know” as the NSFG recorded only six women who refused to provide a response during the entire analytical time period.) Following previous research, we operationalize these latter categories as uncertain fertility intentions (goal uncertainty). Given the construction of the question and responses, *goal uncertainty* is likely underestimated in the NSFG (and other similar fertility surveys), and our estimates should be interpreted as measuring lower bounds.

Respondents who intend any children^3^ are asked how *certain* they are that they will have another child, with possible answers “very sure”, “somewhat sure”, and “not at all sure”. Specifically, they are asked “Of course, sometimes things do not work out exactly as we intend them to, or something makes us change our minds. In your case, how sure are you that you (and [name of current husband or cohabiting partner] if partnered) will have a/nother baby (after this pregnancy is over)? Would you say very sure, somewhat sure, or not at all sure?” This question explicitly recognizes that planned and imagined futures might not be realized and/or that individuals might change their intentions, representing the dimension of uncertainty we characterize as *realization uncertainty*.

In addition to goal uncertainty and realization uncertainty, we consider the *intensity* of goals. The NSFG includes a few attitudinal measures to provide a broad overview on the general childbearing and family social landscape. Of particular relevance for our purposes, respondents are asked “If it turns out that you do not have any children, would that bother you a great deal, some, a little, or not at all?” We created a binary measure equal to 1 if respondents would be “not at all bothered” or “a little bothered” (not intense intentions) and 0 if respondents would be “some bothered” or “a great deal bothered” (intense intentions). Before 2015, this question was asked only of childless women; because we are interested in trends over time, we limit our analyses of intensity to childless women.

As a summary, Table 1 provides the definition of goal uncertainty, realization uncertainty, and intensity of goals and the operationalization used in our study.

**Table 1:** Summary table providing the definition and operationalization of goal uncertainty, realization uncertainty, and intensity of goals

Measure	Definition	Operationalization in the national survey of family growth
Goal uncertainty	Uncertainty about goals themselves, i.e., what is desired or intended. It has been operationalized as “don’t know”, “probably yes/probably no”, “not sure”, and “up to God” answers to fertility goals questions	“Don’t know” answers to the following question: “Do you (and [name of current married or cohabiting partner] if partnered) intend to have (a/nother) baby at some time (in the future/after this pregnancy is over)?”
Realization uncertainty	Uncertainty about realizing fertility goals, that is, when individuals have specific goals to have (or not have) children but are not sure whether they will achieve their goals given their evaluation of their current and future circumstances	“Of course, sometimes things do not work out exactly as we intend them to, or something makes us change our minds. In your case, how sure are you that you (and [name of current husband or cohabiting partner] if partnered) will have a/nother baby (after this pregnancy is over)? Would you say very sure, somewhat sure, or not at all sure?”
Intensity of goals	The intensity with which individuals hold convictions or strong feelings about achieving their goals to have (or not have) children	“If it turns out that you do not have any children, would that bother you a great deal, some, a little, or not at all?”

### Timing and quantum of fertility intentions

We also assess how overall fertility goals are associated with specific goals related to quantum and timing. Respondents who intend more children are asked how many additional children they intend (quantum) and, starting from the 2012 data release, when they intend the next child (timing). For the latter, response categories include “within the next 2 years”, “2–5 years,” “more than 5 years from now,” and “don’t know.” We collapse this into “less than two years” and “more than two years” to distinguish between respondents who intend to have a child soon and respondents who intend to postpone their fertility intentions. “Don’t know” answers about timing (42 women, 0.38% of the sample) are categorized as “more than 2 years.” An alternative categorization into “less than 5 years” and “more than 5 years” shows similar results (not shown).

### Covariates

Fertility and fertility intentions vary by key life course stages and socioeconomic status (Guzzo & Hayford, 2020). In the absence of longitudinal data to track changes in uncertainty across the life course, we consider three specific indicators of life course stage: age (disaggregated into < 30 and 30 +), parenthood status (childless vs. parents, including those who are currently pregnant at the time of interview), and partnership status (partnered via cohabitation or marriage vs. unpartnered/no coresidential partner—importantly, the NSFG only directly measures coresidential partnerships with different-sex partners). We consider two specific indicators of socioeconomic status: education (less than a Bachelor’s degree vs. Bachelor’s degree or more)^4^ and household income as a percentage of the poverty level (below 200% of the poverty level vs. above or at 200% of the poverty level; this variable is computed directly by the NSFG, and an alternative threshold, household income below 100% of the poverty level, shows similar results).

### Analytical strategy

Given our goal of providing a broad picture of multiple dimensions of uncertainty and intensity of fertility intentions in the U.S., we present several largely descriptive analyses. First, we estimate (cross-sectional) levels and trends of goal uncertainty, realization uncertainty, and intensity among women aged 15–44 from 2002 to 2018. We provide aggregate and stratified estimates by the key covariates to assess whether and how these forms of uncertainty are unevenly distributed across life course and socioeconomic status characteristics and whether individuals in those statuses exhibit different trends.

Second, we analyze whether realization uncertainty and intensity are (independently) associated with the reported quantum and timing of fertility intentions; we do not consider variation in goal uncertainty because, to preview the results below, very few women reported “don’t know” fertility intentions. In particular, we estimate the number of additional intended children and the adjusted probability of intending the next child in less than 2 years across realization uncertainty (for individuals who are “very sure”, “somewhat sure”, and “not at all sure”) and across intensity (individuals who would be “bothered”—intense—or “not bothered”—not intense—if they did not have children) using OLS and logistic regressions, respectively. Doing so provides insight into how overall uncertainty may translate into particular behavioral aspects, to the extent that overall uncertainty is linked to fertility postponement and reductions in intended parity.

In all regressions, we control for the life course and socioeconomic status characteristics discussed above as well as additional socio-demographic characteristics that may be associated with both uncertainty and intended quantum and tempo of fertility. Potential confounders that we control for include: race-ethnicity (non-Hispanic White, non-Hispanic Black, Hispanic, Other), a dichotomous indicator of employment status (any employment vs. none), residential area (principal city of metropolitan statistical area, other city of metropolitan statistical area, and not metropolitan statistical area, according to respondent’s address and the U.S. Office of Management and Budget metropolitan statistical areas), religiosity (a 1–3 scale about the importance of religion for respondents, where higher scores equate to more importance), and NSFG data release year. These regression analyses aim to investigate and describe associations rather than provide causal estimates. We compute estimates first among childless women who intend more children to include both realization uncertainty and intensity. We then examine realization uncertainty among all women who intend more children (as intensity is measured only among childless women for most survey years). All analyses and regressions are weighted to provide nationally representative estimates.

Data processing, modeling, and visualizations were carried out in R (RStudio Team 2020) and Stata (StataCorp 2023). Survey weights have been applied using the *srvyr* package in R and the command *svyset* in Stata.

Table 2 reports sample sizes for the various NSFG data releases and weighted descriptive statistics of the main variables.

**Table 2:** Sample size by data release and weighted summary statistics

Variable	Sample size/proportions/mean
NSFG data release	
2002	7,641
2008	12,269
2012	5,599
2014	5,698
2016	4,877
2018	5,408
Fertility intentions	
Don’t know	0.02
No	0.26
Yes	0.72
Realization uncertainty (among women who intend more children):	
Not at all sure	0.07
Somewhat sure	0.36
Very sure	0.57
Intensity (among childless women who intend children):	
Not intense	0.23
Intense	0.77
Number of additional intended children (mean)	1.98
Timing of positive fertility intentions	
< 2 years	0.24
≥ 2 years	0.76
Parity	
0	0.44
1	0.17
2	0.21
3	0.12
4 +	0.06
Age group	
15–29	0.50
30–44	0.50
Partnership status	
Not partnered	0.47
Partnered	0.53
Education	
Less than high school diploma	0.20
High school diploma	0.24
Some college or associate degree	0.30
Bachelor’s degree or more	0.26
Poverty level income	
0–99%	0.24
100–199%	0.22
200–299%	0.17
300–399%	0.13
400–499%	0.10
500% or more	0.14
Employment status	
Employed	0.68
Not employed	0.32
Race and ethnicity	
Non-Hispanic White	0.61
Hispanic	0.17
Non-Hispanic Black	0.15
Other	0.06
Residential area	
Principal city of Metropolitan Statistical Area	0.37
Other city of Metropolitan Statistical Area	0.47
Not Metropolitan Statistical Area	0.16
Religiosity (scale 1–3, from “not important” to “very important”)	2.18

NSFG data (2002–2019). *N* = 41,492 women aged 15 to 44

## Results

### Levels, trends, and variation in goal uncertainty, realization uncertainty, and intensity

**Fig. 2:**
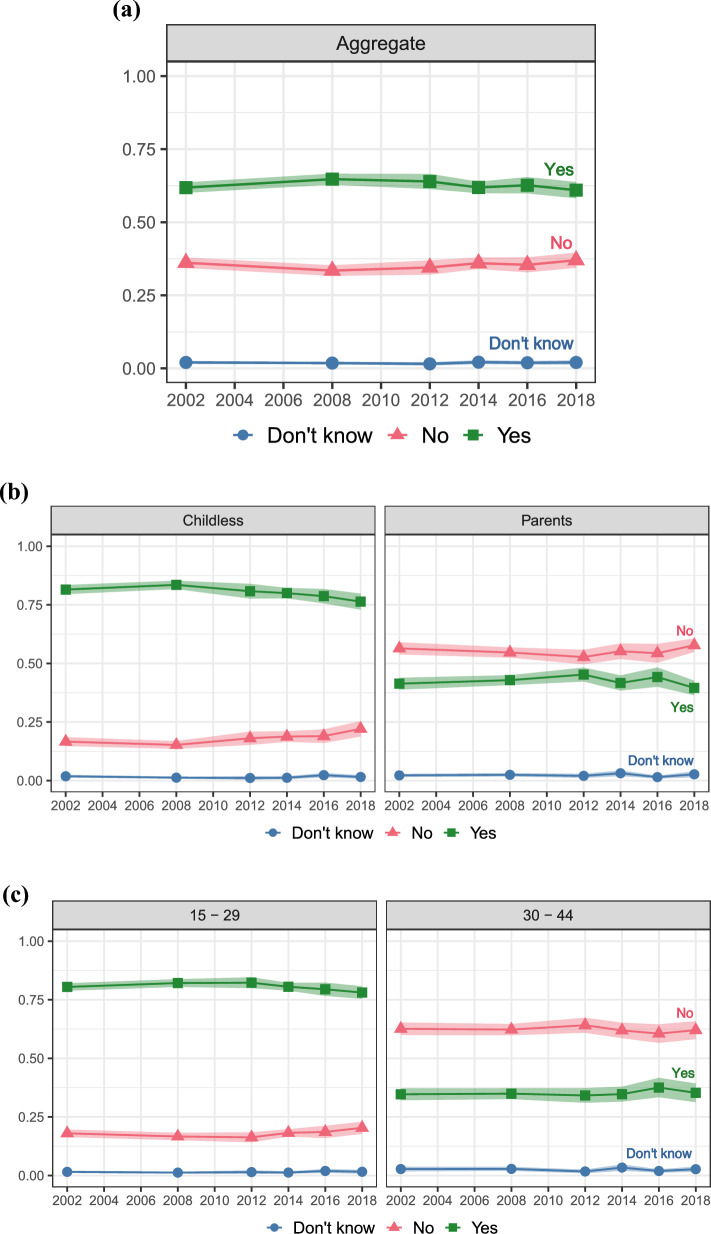

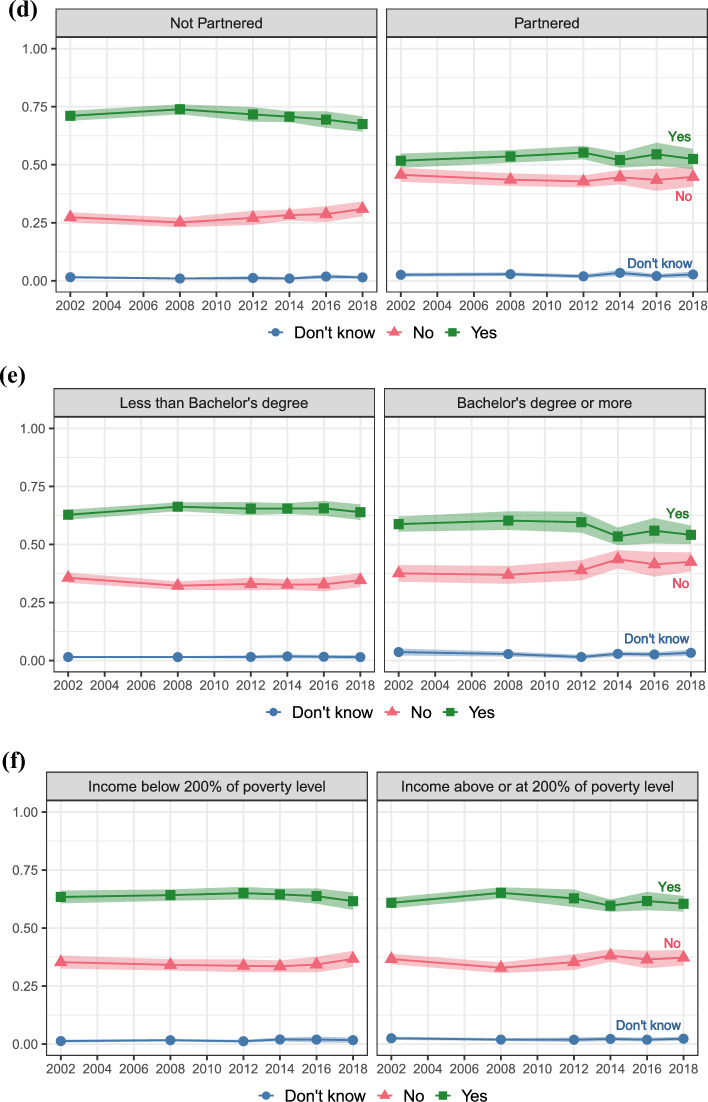
Trends in intentions to have a(nother) child; proportions of *don’t know*, *no*, and *yes* answers with 95% Confidence Intervals. Respondents who are (and their partners are, if any) physically able to have children. Aggregate level (panel **a**), stratified by parental status (panel **b**), by age (panel **c**), by partnership status (panel **d**), by education (panel** e**), and by income poverty level (panel **f**)

First, we present results for goal uncertainty, uncertainty about fertility intentions themselves. Figure 2 shows trends in intentions to have a(nother) child. The top panel (a) shows aggregate proportions, while the subsequent panels show proportions stratified by parental status (panel b), age (panel c), partnership status (panel d), education (panel e), and income poverty level (panel f). The solid blue line (circle markers) represents the proportion of those who don’t know whether they intend more children, the red line (triangle markers) those who do not intend more children, and the green line (square markers) represents those who intend more children. The shaded area represents the 95% confidence intervals. The proportion of “don’t know” answers represents individuals who are uncertain about their fertility intentions.

Across the study period, from 2002 to 2018, we observe few changes in the cross-sectional proportion of women who intend, do not intend, and are unsure about having a(nother) child, despite observed fertility declines following the Great Recession. On average, about 62% of women intend to have more children, 35% do not intend to, and only a minority of women report “don’t know” answers. These “don’t know” answers are stable across the study period and did not significantly increase following the Great Recession, ranging from a minimum of 1.5% in 2012 to a maximum of 2.1% in 2014. The relatively small proportion of women reporting “don’t know” answers to fertility intentions questions aligns with previous studies in the U.S. (e.g., Morgan, 1981) and in other high-income contexts (e.g., Comolli, 2023) but could also be an artifact of the survey wording and response categories. Although the proportions of women who intend and do not intend to have another child vary significantly (as expected) by key life course characteristics, goal uncertainty does not vary significantly and is similar across age groups, parental and partnership status, education, and household income.

Our primary focus is on uncertainty, not on trends in the proportion of respondents who intend more children. Still, it is interesting to note that estimates stratified by life course and socioeconomic status reveal some important trends in intentions. For instance, starting from the aftermath of the Great Recession, there is a decline in the proportion of childless women who intend children (from 84% in 2008 to 76% in 2018), younger women who intend more children (from 82% in 2008 to 78% in 2018), and unpartnered women who intend more children (from 74% in 2008 to 68% in 2018). More recently, there is a decline in the proportion of highly educated women who intend more children (from 60% in 2014 to 54% in 2018). Similarly, there is a symmetrical increase in the proportion of women who do not intend more children among these subgroups.

**Fig. 3:**
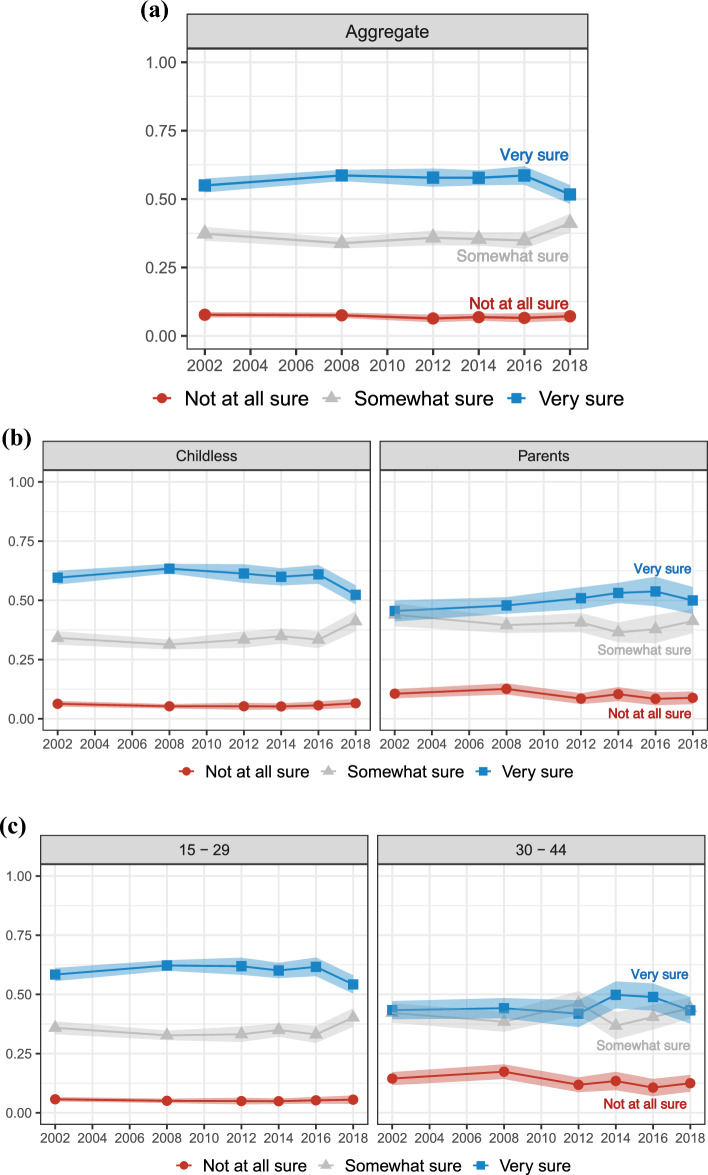

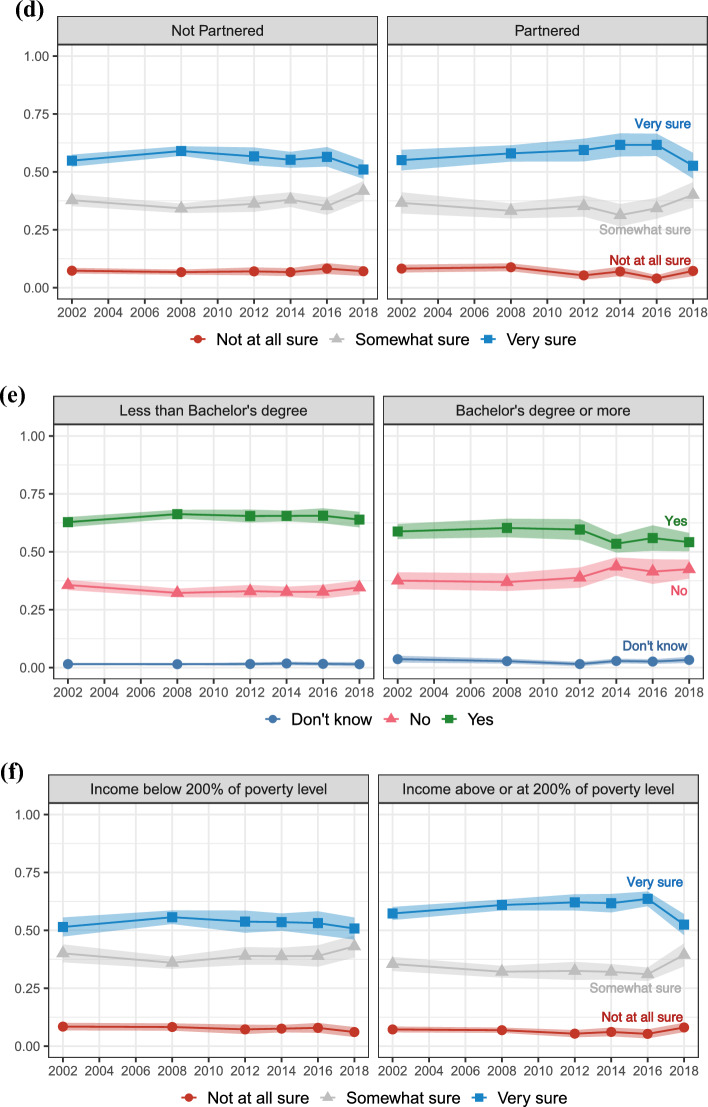
Trends in uncertainty of having a(nother) child when intending a(nother) one; proportions of *not at all sure*, *somewhat sure*, and *very sure* answers with 95% Confidence Intervals. Aggregate level (panel **a**), stratified by parental status (panel **b**), age (panel **c**), partnership status (panel **d**), education (panel **e**), and income poverty level (panel **f**)

Second, we present results for realization uncertainty, uncertainty about realizing positive fertility intentions. Figure 3 shows the perceived realizability of fertility intentions among women who intend to have more children. Again, the top panel (a) shows aggregate proportions, while the subsequent panels show proportions stratified by parental status (panel b), age (panel c), partnership status (panel d), education (panel e), and income poverty level (panel f). The red lines (circle markers) represent those who intend more children but are “not at all sure” they will have more children, the grey lines (triangle markers) those who intend more children and are “somewhat sure” they will have more children, and the blue lines (square markers) women who intend more children and are “very sure” they will have more children. Women responding “very sure” represent those who report *certain* (positive) intentions to have more children and are also *certain* about realizing their intentions. Conversely, women responding “somewhat sure” or “not at all sure” answers, despite reporting *certain* (positive) intentions to have more children, are *uncertain* about realizing their intentions.

According to this definition, we observe a high level of realization uncertainty, with up to 50% of women being uncertain about realizing their intentions to have more children. In particular, about 7% of women between 15 and 44 who intend more children are “not at all sure” they will follow up with their intentions and have more children. Similar to the trend of “don’t know” responses, the uncertainty of realizing positive fertility intentions is overall stable across the study period. In the last 2018 data release, we observe a significant decline in the proportion of “very sure” answers and a significant increase in “somewhat sure” answers, but more data points would be necessary to establish this trend.

Next, we consider how realization uncertainty may vary by key life course statuses and stages. Looking first at parental status, panel b shows that childless respondents who intend to have children are more certain they will have children than parents who intend more children. About two-thirds of childless women are certain they will have at least one child, in contrast to about one-half of mothers who are certain about having at least one more child. Across the study period, mothers who intend more children show a slight increase in the certainty of meeting their positive intentions, while childless women show a slight decrease. In 2002, 46% of mothers and 60% of childless women reported “very sure” answers, while in 2018, this was the case for 50% of mothers and 52% of childless women.

There are clear age differences in the certainty of meeting intentions to have more children. Panel c shows that women between 15 and 29 who intend more children are more certain of realizing their intentions than women between 30 and 44. For instance, more than 50% of women between 30 and 44 who intend more children are uncertain about realizing them, with up to 17% of women reporting being “not at all sure” in 2008. Still, about one-third of women between 15 and 29 who intend more children are also uncertain about realizing their intentions.

Panel d shows that partnered women who intend more children are slightly more certain of realizing their intentions than unpartnered women. Consistent with the aggregate proportions, there is no clear time trend following the Great Recession but overall stability.

Panel e and panel f also show socioeconomic differences in realization uncertainty. Women with at least a Bachelor’s degree and those with an income at or above 200% of the poverty level are slightly more certain of realizing their intentions than women with less than a Bachelor’s degree and those with an income below 200% of the poverty line. Still, recent trends suggest that the certainty of realizing positive fertility intentions is declining among high socioeconomic status women. The proportion of women with a Bachelor’s degree who intend more children and are “very sure” they will have more children declined from 65% in 2014 to 54% in 2018, and the proportion who are “not at all sure” reached 9%, equating the peak of 9% observed during the Great Recession in 2008. Generalized uncertainty about economic conditions in the United States may be affecting even those with relatively more resources.

**Fig. 4:**
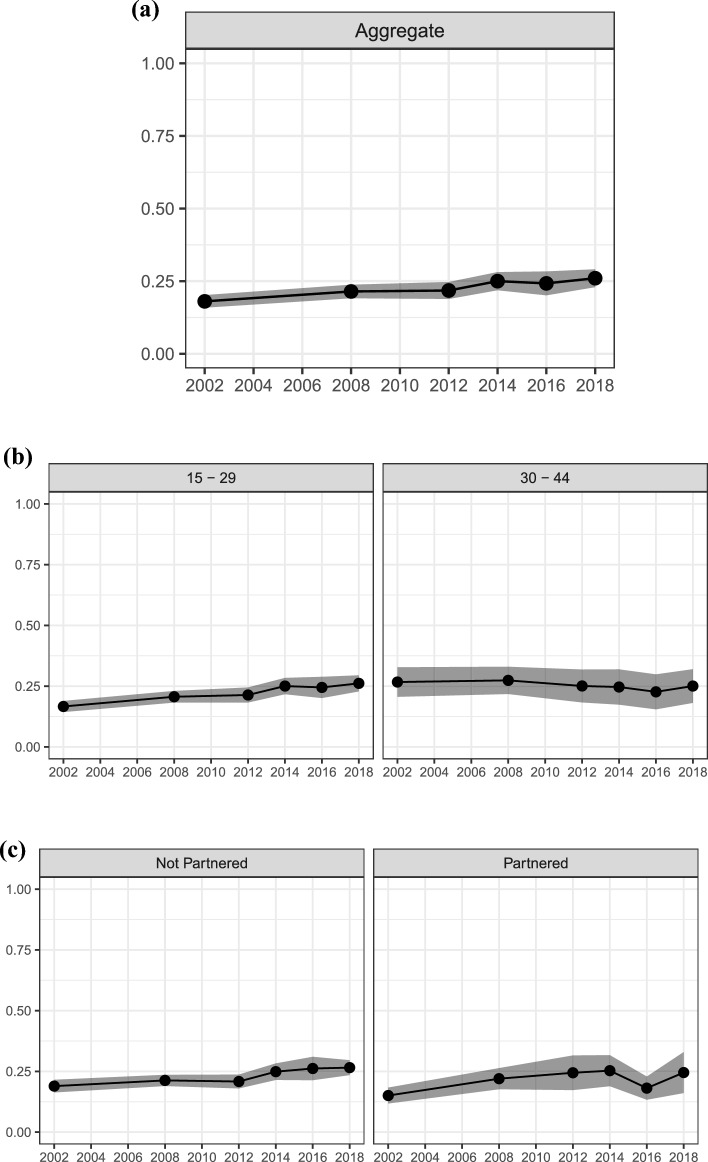

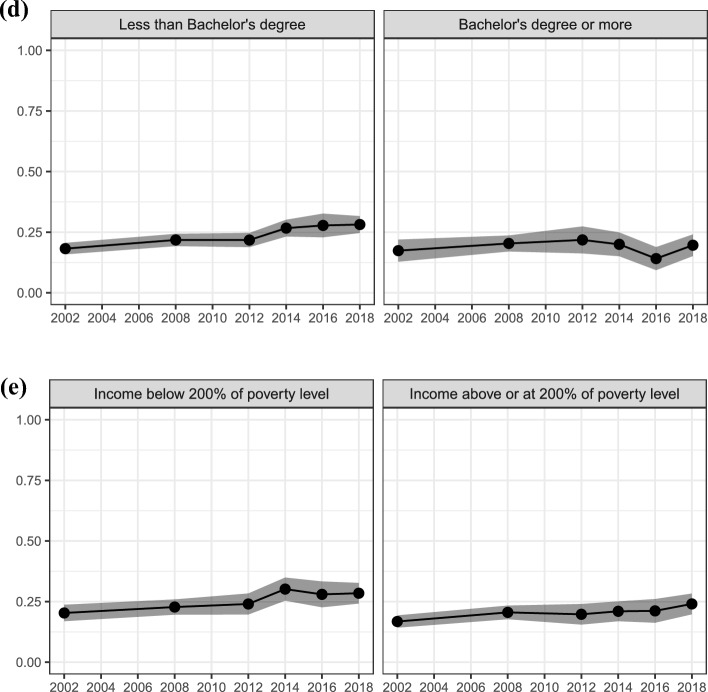
Trends in fertility intentions intensity (being bothered if it turns out to not have any children when intending) among childless women who intend one or more children; proportions of *not at all bothered* or *a little bothered* answers with 95% Confidence Intervals. Aggregate level (panel **a**), stratified by age (panel **b**), by partnership status (panel **c**), by education (panel **d**), and by income poverty level (panel **e**)

Third, we present results for intensity of intentions, operationalized as the proportion of women who intend more children and would be “not at all bothered” or “a little bothered” if they did not have any children. As described above, we focus on childless women who intend to have children. Figure 4 reports the intensity of intentions among childless women who intend to have at least one child. The top panel (a) shows aggregate proportions, while the subsequent panels show proportions stratified by age (panel b), partnership status (panel c), education (panel d), and income poverty level (panel e). The dashed lines represent the 95% confidence intervals.

Results show relatively high and increasing levels of women with low intensity of intentions. In 2002, 18% of childless women who intended to have at least one child reported weak intentions, while in 2018, this was the case for 26% of women, a 44% increase. As shown in panel b, the increase in low intensity intentions is driven by young women aged 15 to 29. In 2002, 17% of childless women between 15 and 29 reported low intensity, compared to 27% of childless women between 30 and 44. In 2018, 26% of childless women between 15 and 29 reported low intensity, similar to the 25% of those between 30 and 44. Panel c shows limited differences by partnership status, and that low intensity of intentions is increasing both among partnered and not partnered women. On the contrary, panel d and e show that women with less than a Bachelor’s degree and those with an income below 200% of the poverty level report a weaker intensity of intentions. In 2018, 28% of women with less than a Bachelor’s degree reported low intensity, compared to 20% of women with a Bachelor’s degree or more. Similarly, in 2018, 28% of women with an income below 200% of the poverty level reported low intensity, compared to 24% of women with an income above or at 200% of the poverty level.

### Realization uncertainty, intensity, quantum and timing of fertility intentions

**Fig. 5:**
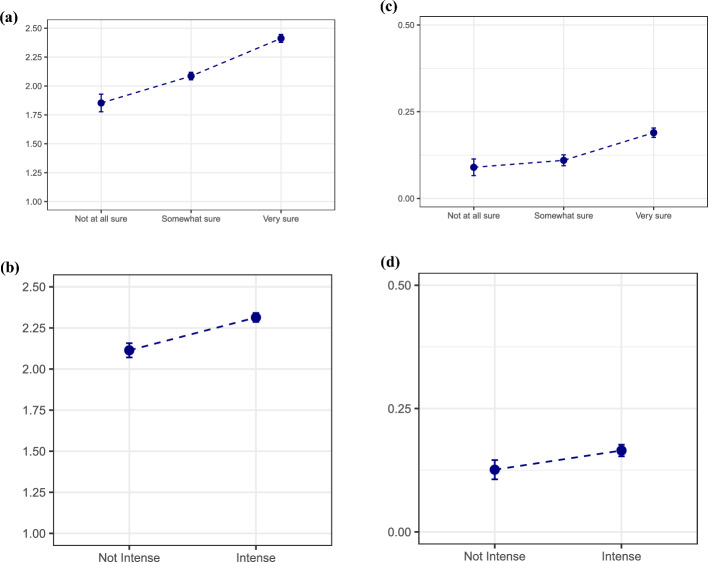
Post-estimation estimates of weighted regressions (OLS and logistic regression) predicting the number of additional intended children (upper panel) and the intended timing of the next child (Less than two years Vs. More than two years) (lower panel) among childless women who intend to have children by realization uncertainty and intensity. Controls: Age, partnership status, race and ethnicity, education, employment status, poverty level income, residential area, religiosity, and NSFG release. See Tables 3 and 4 for regression estimates

To continue, we analyze whether realization uncertainty and intensity of intentions are independently associated with the quantum and timing of fertility intentions, net of several life course and socioeconomic status characteristics. Figure 5 shows the association between realization uncertainty and intensity with the number of additional intended children and the intended timing of the next pregnancy for childless women who intend more children. The upper panels show the predicted number of additional intended children, while the lower panels show the predicted probability of intending the next child in less than two years. Controls for age, parity, race and ethnicity, education, employment status, poverty level income, residential area, religiosity, and NSFG release are included in the regression models. Regression estimates are reported in Tables 3 and 4 (Appendix).

Figure 5 shows that women who are more certain about realizing their intentions have a higher intended parity, as do those who have more intense intentions about having children. They are similarly associated with a shorter intended time frame for their (next) birth. On average, after controlling for key life course and socioeconomic characteristics, childless women who are “very sure” about meeting their intentions report intending 2.4 children, those who are “somewhat sure” 2.1 children, and those who are “not at all sure” 1.9 children. Similarly, childless women who would be bothered if they did not achieve their intentions of having children report intending 2.3 children, while those who would not be bothered 2.1 children. Looking at the intended timing, the adjusted predicted probability of intending the next child in less than two years is 19% for those who are “very sure” about realizing their intentions to have more children, 11% for those who are “somewhat sure,” and 9% for those who are “not at all sure.” Similarly, it is 16% for women who have more intense intentions and 13% for those who feel less strongly.

**Fig. 6:**
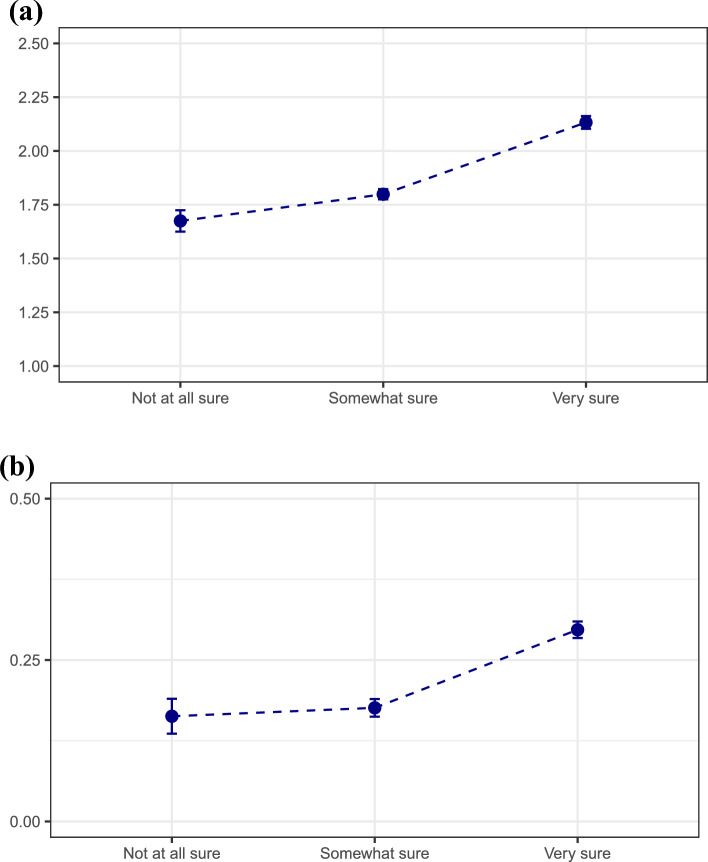
Post-estimation of weighted regressions (OLS and logistic regression) predicting the number of additional intended children (panel** a**) and the intended timing of the next child (Less than 2 years vs. More than 2 years) (panel** b**) among women who intend more children. Controls: Age, parity, partnership status, race and ethnicity, education, employment status, poverty level income, residential area, religiosity, and NSFG release

These analyses show that both realization uncertainty and intensity of intentions are clearly associated with the quantum and timing of fertility intentions after controlling for key characteristics related to individuals’ agency and life course statuses. Analyses about realization uncertainty including all women who intend more children show similar and robust results (Figure 6).

## Discussion and conclusion

In this paper, motivated by a macro–micro framework that links macro-level uncertainty to fertility behaviors through the uncertainty in fertility goals and a growing understanding of the multidimensional nature of fertility goals, we provide a broad descriptive picture of trends and patterns in three dimensions of uncertainty related to fertility intentions and attitudes in the U.S. Using data from the NSFG from 2002 through 2018, we map overall trends and trends by age, parity, partnership status, education, and income as a percentage of poverty level in uncertainty about goals themselves (goal uncertainty), uncertainty about whether goals will be realized (realization uncertainty), and variation in the intensity of intentions (as operationalized by whether women would be bothered by not fulfilling their intentions).

Overall, we found stability in the cross-sectional proportion of women who intend to have more children, do not intend, and answer “don’t know” to fertility intentions questions. In the NSFG, where alternative uncertain categories such as “probably not” and “probably yes” are not provided as answers to fertility intentions questions and “don’t know” answers are not explicitly provided (see Data and Methods section), only a small minority of women report “don’t know” fertility intentions directly. This suggests that the first kind of uncertainty—uncertainty about the goals themselves—is relatively rare, though this is a lower bound estimate. Nevertheless, up to 50% of women who intend to have at least one (more) child are uncertain whether they will realize their goals; that is, realization uncertainty is quite common. Further, our analyses provided evidence of variation in the intensity with which intentions are held. We found that among childless women who intend a child, a substantial share (up to 25%) report they would not be bothered if they did not have a child. These findings suggest that despite reporting positive intentions to have a child, many women may not have strong feelings about having a child. This complements previous research showing that also childless men are increasingly more likely to report a disinterest in childbearing and being not bothered if they did not have a child (Bozick, 2023).

Across the study period, our analyses show high but overall stable levels of goal and realization uncertainty and declining levels of intensity. These patterns are particularly apparent among childless and young women, possibly indicating cohort changes for women at the beginning of their childbearing careers. Despite aggregate stability in goal uncertainty, we found important trends across population subgroups that might anticipate future fertility changes. Starting from the aftermath of the Great Recession, we found a decline in the proportion of childless, young, and unpartnered women who intend more children, and a recent decline also among highly educated women. These results are consistent with previous research suggesting that the negative impact of the Great Recession has been particularly salient among young and childless women, who might have revised downward their fertility intentions.

Our results highlighted important differences in realization uncertainty and intensity across life course and socioeconomic status characteristics. Parents and older respondents who intend more children are less certain of realizing their intentions and hold their intentions less intensely. On the contrary, women with a Bachelor’s degree and those with an income above or at 200% of the poverty level are more certain about realizing their intentions and hold their intentions more intensely, although recent trends suggest certainty and intensity are declining for high socioeconomic status respondents as well. These analyses show that constraints previously associated with downward revisions of positive fertility intentions (Iacovou and Tavares 2011), gaps between fertility goals and behaviors (Morgan & Rackin, 2010; Régnier-Loilier and Vignoli 2011; Spéder and Kapitány 2009), and gaps between fertility desires and intentions (Badolato, 2025), are also associated with higher uncertainty among women who intend more children.

Finally, we showed that, among women who intend to have more children in the future, those who are certain of realizing their positive intentions and hold intentions intensely report a higher number of additional intended children and a shorter time frame for future childbearing, net of several sociodemographic characteristics. Put differently, our findings suggest that increased realization uncertainty and reduced intensity are likely part of a ‘package’ of fertility goals wherein more certainty and intensity are linked to a tighter timeline for childbearing and a greater number of intended children. More specific measures of fertility goals, like those that include shorter time frames, are more predictive of subsequent fertility (Dommermuth et al., 2015). Thus, our findings suggest that the high levels of realization uncertainty and low levels of goal intensity may contribute to declining fertility, even when intentions themselves are stable.

### Limitations

Like others studying fertility goals with secondary data, we are constrained by the available survey questions in our analyses. Different available measures (such as “probably yes” and “probably no” options or questions about pregnancy avoidance) will lead to slightly different operationalizations (Miller et al., 2013; Weitzman et al., 2017). The lack of a specific “don’t know” response for intentions, in particular, likely underestimates the true share of goal uncertainty in our analyses. Our analysis is motivated by previous research finding growing macro-level uncertainty across multiple domains in the United States. In this initial descriptive analysis, we do not directly measure macro-level uncertainty or its expression in individual lives. The NSFG contains only basic measures of individual characteristics, with no measures of psychological traits or outlooks and no measures of family-related attitudes (not available after the 2008 data release). Contextual indicators of economic conditions and uncertainty are not publicly available. We thus limit our analysis of variation to key life course and socioeconomic measures. As anticipated in the theoretical background, it is also possible that individuals who desire larger families and intend to have children in the near future might be more certain of realizing their intentions and hold them more intensively. Because of differences in the way data are collected for men and women in the NSFG, we are unable to conduct analyses of uncertainty in men’s fertility goals. We also have limited information on the goals of male partners of women in the sample. Finally, the NSFG has been designed to be nationally representative, and state-level estimates of uncertainty in fertility goals cannot be computed. Our analysis of women’s goals constitutes an important first step but does not provide a full picture of how Americans envision childbearing. Future research should extend our findings by exploring the role of contextual indicators of economic conditions and uncertainty, the interplay between the quantum and timing of fertility goals and uncertainty, and by linking the uncertainty in fertility goals and the timing and quantum of goals to subsequent behaviors. To do so, longitudinal data with detailed information on both contextual economic conditions and perceived uncertainty and fertility goals are necessary.

### Conclusion

Together, our findings show the multidimensionality of fertility intentions uncertainty and the importance of a unified framework that links fertility trends, fertility intentions uncertainty and intensity, and the quantum and tempo of fertility goals. Uncertainty in childbearing goals may reflect broader uncertainty about achieving stable lives and are likely linked to economic turmoil; other factors, such as worries about discrimination linked to gender identity and sexual orientation, limitations in access to reproductive health care, and ongoing political and civic disruption in the U.S. and elsewhere may also play a role (e.g., Gustafson, Manning, and Kamp Dush 2025; Perelli-Harris et al., 2024). These changes result in what could be considered *institutionalized uncertainty*, uncertainty created by institutions that, in turn, affect the uncertainty of childbearing decisions.

Although the cross-sectional proportion of individuals who intend to have a(nother) child has been stable since the Great Recession, we found evidence of declining intentions among young and childless women, and the major socioeconomic and institutional changes that have occurred in its aftermath are likely creating a broader climate of uncertainty. This uncertainty, which exists across multiple domains, seems to be related to childbearing decisions and behaviors—and the link between the two. If a substantial share of women of childbearing age are unsure if they will realize their fertility goals and an increasing proportion do not feel particularly motivated to pursue these goals, then fertility rates are likely to continue to decline as long as barriers to achieving goals remain high.

## Data Availability

National Survey of Family Growth data used for this study are publicly available and can be downloaded from the following link: https://www.cdc.gov/nchs/nsfg/index.htm.

## Appendix

See Tables 3, 4 and Fig. 6

**Table 3:** Weighted OLS regression estimates predicting the number of additional intended children for childless women (left columns) and all women (right columns) who intend to have more children

	Childless women		All women	
	Coef	Std. Err	Coef	Std. Err
Realization Uncertainty (ref. Not sure at all)				
Somewhat sure	0.23***	0.04	0.12***	0.03
Very Sure	0.56***	0.04	0.46***	0.03
Intensity (ref. Less intense)	0.20***	0.02	–	–
Age	− 0.04***	0.002	− 0.03***	0.002
Parity (ref. 0 children)				
1	–	–	− 0.64***	0.02
2	–	–	− 0.69***	0.03
3	–	–	− 0.75***	0.03
4 or more	–	–	− 0.44***	0.08
Partnership status (ref. Not partnered)	− 0.14***	0.03	− 0.11***	0.02
Race and ethnicity (ref. NH white)				
Hispanic	0.04	0.03	− 0.03	0.03
Non-Hispanic Black	− 0.15***	0.04	− 0.17***	0.03
Other	− 0.04	0.04	− 0.02	0.03
Education (ref. Less than high school diploma)				
High school diploma	0.11***	0.04	0.09***	0.03
Some college or associate degree	0.26***	0.03	0.20***	0.03
Bachelor’s degree or more	0.32***	0.04	0.25***	0.03
Employed (ref. Not employed)	0.06**	0.02	0.15***	0.02
Income poverty level (ref. 0–99%)				
100–199%	− 0.01	0.03	0.01	0.02
200–299%	0.04	0.03	0.04	0.02
300–399%	0.03	0.04	0.02	0.03
400–499%	− 0.02	0.04	− 0.01	0.03
500% or more	− 0.004	0.04	− 0.01	0.03
Residential area (ref. Principal city of Metropolitan Statistical Area)				
Other city of Metropolitan Statistical Area	− 0.02	0.03	− 0.015	0.02
Not Metropolitan Statistical Area	− 0.05	0.04	− 0.07*	0.03
Religiosity	0.17***	0.02	0.15***	0.01
NSFG data release (ref. 2002)				
2008	0.06	0.05	0.02	0.04
2012	0.04	0.04	0.02	0.04
2014	0.03	0.03	− 0.01	0.03
2016	0.07	0.05	0.02	0.04
2018	0.07*	0.03	0.03	0.03
Intercept	2.02***	0.07	2.27***	0.05
**N**	**13,565**		**20,672**	

The bold values are referring to sample size (N)

Estimated coefficients and standard errors. NSFG data (2002–2019): women aged 15–44 who intend more children

Significance levels: **p* < 0.05, ***p* < 0.01, ****p* < 0.001

**Table 4:** Weighted logistic regression estimates predicting the intended timing of the next child (Less than two years vs. More than two years) for childless women (left columns) and all women (right columns) who intend to have more children

	Childless women		All women	
	OR	95% CI	OR	95% CI
Realization uncertainty (ref. Not sure at all)				
Somewhat sure	1.41	0.88–2.26	1.15	0.84–1.57
Very sure	3.96***	2.48–6.30	3.31***	2.40–4.56
Intensity (ref. Less intense)	1.66***	1.22–2.27	–	–
Age	1.28***	1.24–1.32	1.23***	1.21–1.26
Parity (ref. 0 children)				
1	–	–	1.51***	1.22–1.87
2	–	–	0.81	0.59–1.11
3	–	–	0.72	0.47–1.11
4 or more	–	–	0.65	0.33–1.29
Partnership status (ref. Not partnered)	7.94***	6.11–10.31	4.62***	3.83–5.58
Race and ethnicity (ref. NH white)				
Hispanic	1.88***	1.31–2.68	1.27*	1.01–1.61
Non-Hispanic Black	2.81***	1.96–4.03	1.59***	1.27–1.98
Other	1.49	0.80–2.77	1.04	0.69–1.55
Education (ref. Less than high school diploma)				
High school diploma	0.67	0.40–1.14	0.79	0.57–1.08
Some college or associate degree	0.61*	0.38–0.99	0.68*	0.49–0.93
Bachelor’s degree or more	0.40***	0.23–0.70	0.53***	0.37–0.76
Employed (ref. Not employed)	1.12	0.79–1.58	1.06	0.88–1.28
Income poverty level (ref. 0–99%)				
100–199%	0.53**	0.35–0.79	0.72**	0.57–0.90
200–299%	0.58**	0.39–0.88	0.88	0.68–1.13
300–399%	0.66	0.40–1.09	0.96	0.70–1.32
400–499%	0.64	0.35–1.16	0.88	0.60–1.29
500% or more	0.65*	0.44–0.97	0.98	0.74–1.30
Residential area (ref. Principal city of Metropolitan Statistical Area)				
Other city of Metropolitan Statistical Area	1.16	0.88–1.53	1.15	0.98–1.36
Not Metropolitan Statistical Area	1.59*	1.02–2.46	1.44	1.14–1.82
Religiosity	1.20*	1.03–1.41	1.12	1.01–1.24
NSFG data release (ref. 2012)				
2014	0.89	0.64–1.24	0.91	0.75–1.10
2016	0.83	0.56–1.24	0.93	0.74–1.17
2018	0.98	0.69–1.39	1.11	0.88–1.40
N	**7,244**		**11,034**	

The bold values are referring to sample size (N)

Estimated Odd Ratios and 95% confidence intervals. NSFG data (2012–2019): women aged 15 – 44 who intend more children

Significance levels: **p* < 0.05, ***p* < 0.01, ****p* < 0.001

## Footnotes

1 The NSFG provides separate data for men and women without other measures or further elaborations on gender identity. Following the NSFG’s usage, we use the term “women” to describe the sample.

2 Previous research focused on the difference between fertility desires and intentions. In Miller’s Traits-Desires-Intentions-Behaviors framework, desires are conceptualized as an intrinsic wish to have (or not have) children while intentions entail a specific plan to pursue an actionable childbearing goal and are thus more influenced by external constraints (Miller 2011). Given our focus on the timing and quantum of fertility intentions, it is beyond the scope of this paper to consider that individuals’ fertility desires and intentions do not always align and we will specifically focus on fertility intentions.

3 Partnered women who do not intend more children are also asked the certainty of their (negative) intentions, but unpartnered women who do not desire more children are not asked whether they intend or do not intend more children and, consequently, how certain they will (not) have more children. This limits further analyses, but, given our focus on women who intend more children, does not affect our analyses.

4 In the U.S., family behaviors diverge sharply based on having a four-year college degree or not (Lundberg, Pollak, and Stearns 2016).
